# Prospective deep phenotyping of choroideremia patients using multimodal structure-function approaches

**DOI:** 10.1038/s41433-020-0974-1

**Published:** 2020-05-28

**Authors:** Ahmed M. Hagag, Andreas Mitsios, Akshay Narayan, Alessandro Abbouda, Andrew R. Webster, Adam M. Dubis, Mariya Moosajee

**Affiliations:** 1 Moorfields Eye Hospital NHS Foundation Trust, London, UK; 2 UCL Institute of Ophthalmology, London, UK; 3 Department of Ophthalmology, Great Ormond Street Hospital for Children NHS Foundation Trust, London, UK

**Keywords:** Hereditary eye disease, Prognostic markers

## Abstract

**Objective:**

To investigate the retinal changes in choroideremia (CHM) patients to determine correlations between age, structure and function.

**Subjects/Methods:**

Twenty-six eyes from 13 male CHM patients were included in this prospective longitudinal study. Participants were divided into <50-year (*n* = 8) and ≥50-year (*n* = 5) old groups. Patients were seen at baseline, 6-month, and 1-year visits. Optical coherence tomography (OCT), OCT angiography, and fundus autofluorescence were performed to measure central foveal (CFT) and subfoveal choroidal thickness (SCT), as well as areas of preserved choriocapillaris (CC), ellipsoid zone (EZ), and autofluorescence (PAF). Patients also underwent functional investigations including visual acuity (VA), contrast sensitivity (CS), colour testing, microperimetry, dark adaptometry, and handheld electroretinogram (ERG). Vision-related quality-of-life was assessed by using the NEI-VFQ-25 questionnaire.

**Results:**

Over the 1-year follow-up period, progressive loss was detected in SCT, EZ, CC, PAF, and CFT. Those ≥50-years exhibited more structural and functional defects with SCT, EZ, CC, and PAF showing strong correlation with patient age (*rho* ≤ −0.47, *p* ≤ 0.02). CS and VA did not change over the year, but CS was significantly correlated with age (*rho* = −0.63, *p* = 0.001). Delayed to unmeasurable dark adaptation, decreased colour discrimination and no detectable ERG activity were observed in all patients. Minimal functional deterioration was observed over one year with a general trend of slower progression in the ≥50-years group.

**Conclusions:**

Quantitative structural parameters including SCT, CC, EZ, and PAF are most useful for disease monitoring in CHM. Extended follow-up studies are required to determine longitudinal functional changes.

## Introduction

Choroideremia (CHM, OMIM 303100) is a rare X-linked progressive chorioretinal dystrophy with an estimated prevalence of 1 in 50,000–100,000 [1, 2]. Patients suffer from nyctalopia and progressive visual field constriction, followed by central vision loss in later age [3]. Choroideremia is caused by mutations in the *CHM* gene (OMIM 303390) encoding Rab-escort protein 1 (REP1), which is involved in lipid prenylation and intracellular protein trafficking. The primary site of pathogenesis remains unknown, but dysfunctional REP1 results in accumulated unprenylated Rabs, with abnormal photoreceptor outer segment phagocytosis and ineffective melanosome transport within the RPE leading to cell death [4]. There is significant intra- and inter-familial clinical variability but no established genotype-phenotype correlations. Over 30% of patients have nonsense mutations [1]. There are no commercially approved treatments available for CHM, with variable results have been reported from adeno-associated virus vector gene therapy clinical trials [5, 6]. Preclinical evidence of nonsense suppression therapy with small-molecule drugs, including ataluren and PTC-414, could represent a future therapeutic approach [7]. Natural history studies of CHM are required to refine clinical trial endpoints and for monitoring disease progression.

Several ophthalmic imaging and functional testing modalities have been employed to characterise the cross-sectional structural and functional changes in CHM patients. Optical coherence tomography (OCT) has assessed the degeneration of the outer retina showing retinal thinning and photoreceptor loss [8]. OCT angiography (OCTA) [9] has shown the ability to detect vascular changes in retinal and choroidal circulations non-invasively in CHM highlighting decreased vascular density [10, 11]. Fundus autofluorescence (FAF) has been also used to monitor the progression of retinal pigment epithelium (RPE) and photoreceptor loss [12]. Visual function changes including visual acuity [13], electroretinography (ERG) [14], perimetry [15], and microperimetry [16] have been mapped independently. Most previous investigations were also performed cross-sectionally or retrospectively. In this study, we combine a wide-range of multimodal imaging and functional testing in a prospective cohort to elucidate the clinical phenotype of CHM and potential structure-function correlations. The study also aims to explore potential endpoints for clinical trials.

## Materials and methods

### Study participants and procedures

Male choroideremia patients with molecularly-confirmed mutations were recruited prospectively at Moorfields Eye Hospital. This prospective observational case series was approved by the national research ethics committee and was conducted in adherence to the tenets of the Declaration of Helsinki. Informed written consent was obtained from all participants. Both eyes from each patient were included and they attended three equally spaced visits over a period of 1 year. At baseline, a detailed medical and ocular history was taken with comprehensive ophthalmic examination. ETDRS best-corrected visual acuity (BCVA) and Pelli-Robson contrast sensitivity (CS) were measured. Patients were divided into 2 groups based on age at baseline: group 1 included patients <50 years-old and group 2 were those ≥50 years-old.

### AngioVue OCT/OCT angiography

**Fig. 1:**
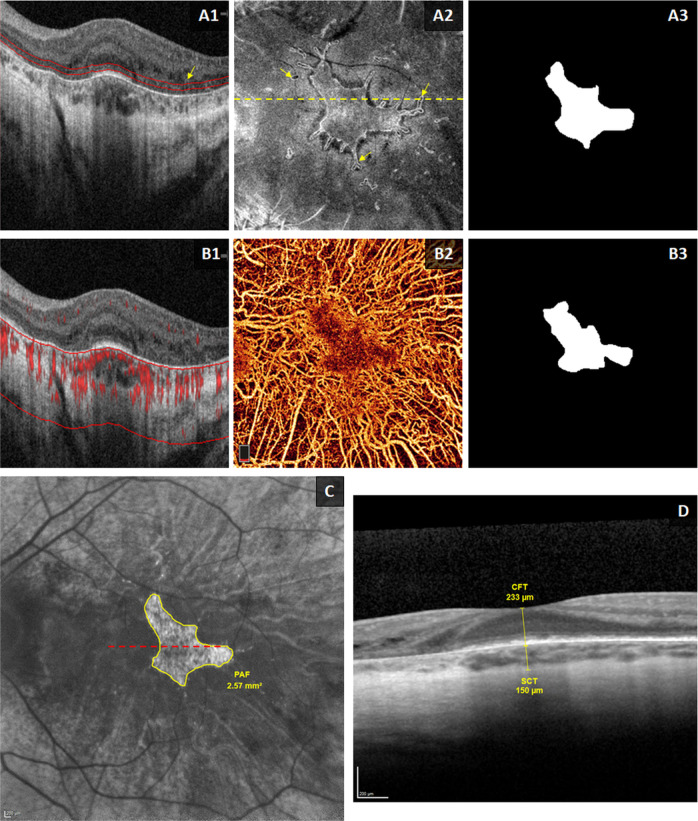
Methods of analysing optical coherence tomography (OCT), OCT angiography (OCTA), and fundus autofluorescence (FAF) images. Images are from the left eye of a 25-year-old male patient with choroideremia. **a1** and **b1** 6-mm cross-sectional structural OCT images from AngioVue (OptoVue) machine. Red signal in **b1** represents the angiographic signal. **a2** *En face* OCT of the ellipsoid zone (EZ) slab, constructed from the mean intensity between the two red lines in **a1** (30–60 μm above Bruch’s membrane [BM]). The yellow dashed line in **a2** corresponds to the OCT B-scan in **a1** and **b1**. Yellow arrows in **a1** and **a2** show outer retinal tubulations. **b2** *En face* OCTA slab of choriocapillaris (CC), generated as the maximum projection of flow signal below the BM (approximate boundaries are represented by the red lines in **b1**. **a3** and **b3** Binary masks of the manually segmented areas of preserved EZ and CC from the *en face* images in **a2** and **b2**, respectively. **c**: 30° FAF image with manual delineation and measurement of the area of preserved AF. Red dashed line corresponds to the location of the Spectralis OCT image in **d**. Central foveal thickness (CFT) was measured between the internal limiting membrane and BM, while the subfoveal choroidal thickness (SCT) was measured between BM and the choroid-sclera interface.

AngioVue (OptoVue, Inc.) 6 × 6 mm (304 A-scan/B-scan, 304 B scans) macular OCT/OCTA scans were acquired. Angiograms were checked on ReVue software for quality and segmentation by an expert grader (AMH). Images with low quality (Q < 6), significant shadowing or motion artefacts were excluded. The boundary of Bruch’s membrane (BM)/RPE was corrected from the structural OCT B-scans and used as reference for subsequent *en face* analysis. *En face* OCT image of the ellipsoid zone (EZ) was constructed from the mean reflectance between 30 and 60 μm above the BM. This slab provides relatively sharp boundaries of the preserved photoreceptors and outer retinal tubulations (ORT) (Fig. 1, a2). *En face* choroidal OCTA was also generated from the maximum flow projection below the BM, displaying the preserved choriocapillaris and the exposed larger choroidal vessels (Fig. 1, b2). The preserved EZ and CC areas were manually delineated from the *en face* EZ and CC slabs using ImageJ (Fig. 1). In scans where more than one continuous island of EZ or CC were detected within the 6 mm area, only the central island was selected and measured. A subset of baseline images was graded repeatedly to assess the intra- and inter-grader variability.

### Spectralis OCT and fundus autofluorescence

The Spectralis HRA + OCT machine (Heidelberg Engineering, Inc.) was used to acquire 20° volumetric OCT scans and 30° FAF images. OCT and FAF images were analysed using the Heidelberg Eye Explorer (HEYEX) software. The central foveal thickness (CFT) and subfoveal choroidal thickness (SCT) were measured. The CFT was measured between the inner limiting membrane (ILM) and BM, and the SCT was measured between the BM and choroid-sclera interface (Fig. 1d) [8]. The central preserved autofluorescence (PAF) area was manually delineated and measured (Fig. 1c) as previously described [12].

### Microperimetry and static perimetry

**Fig. 2:**
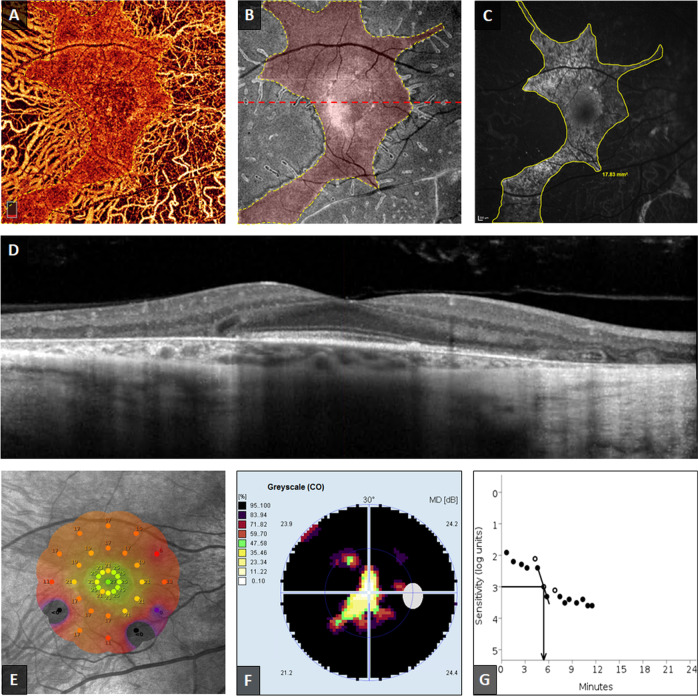
Structural and functional findings from the right eye of a 49-year-old (group 1) patient with choroideremia. **a** Constructed *en face* optical coherence tomography angiogram (OCTA) of the choriocapillaris (CC). **b** *En face* OCT image of the ellipsoid zone (EZ) slab. Preserved areas of CC and EZ are highlighted in red and outlined by yellow dashes in **a** and **b**, respectively. **c** Fundus autofluorescence (FAF) image with area of preserved AF (PAF) outlined in yellow. Very close agreement between areas of CC, EZ, and PAF can be appreciated. The cross-sectional OCT image in **d** corresponds to the red dashed line in **b**, and showing extensive outer retinal and choroidal degeneration in the periphery, with the presence of outer retinal tubulations beyond the relatively preserved central retinal island. **e** MAIA colour-coded mesopic sensitivity map. Reddish colours represent areas with decreased sensitivity, while greenish colours represent more preserved sensitivity. The map demonstrates decreased macular sensitivity especially at the periphery, with dense scotomas at the areas outside the central retinal island (black spots). The Octopus visual field (VF) in F demonstrates the severely constricted VF, with a small island of preserved vision closely related to the shape of preserved retinal tissue. This eye was the only one in our cohort with a normal rod intercept time as measured by dark adaptometry **g**.

**Fig. 3:**
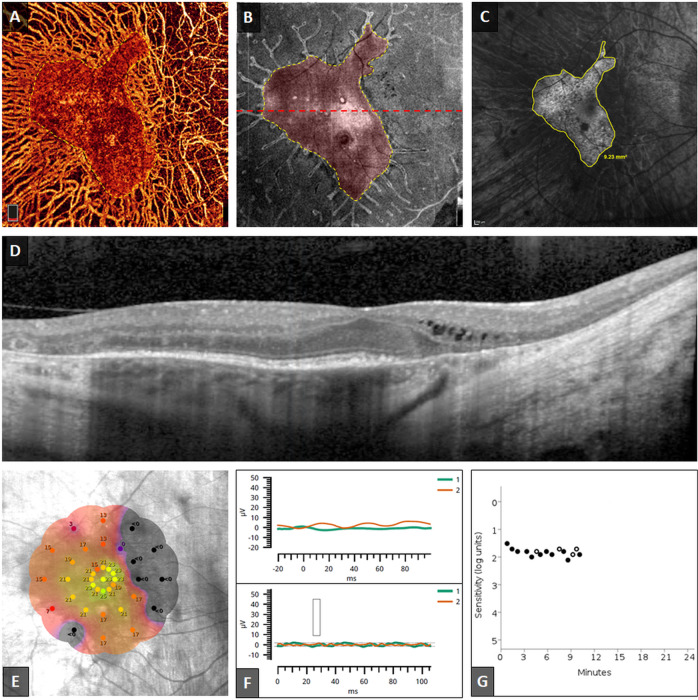
Structural and functional findings from the right eye of a 62-year-old (group 2) patient with choroideremia (CHM). **a***En face* optical coherence tomography angiogram (OCTA) of the choriocapillaris (CC). **b** *En face* OCT image of the ellipsoid zone (EZ) slab. Preserved areas of CC and EZ are highlighted in red and outlined by yellow dashes in **a** and **b**, respectively. **c** Fundus autofluorescence (FAF) image with area of preserved AF outlined in yellow. Relative preservation of retinal tissue can be observed on the temporal side of the fovea, as compared to the more degenerated nasal side. The central retinal island appears significantly smaller in this patient as compared to the younger patient in Fig. 2. The cross-sectional OCT image in **d** corresponds to the red dashed line in **b**. Cystoid macular oedema can be detected outside the nasal edge of the central retinal island. **e** MAIA colour-coded mesopic sensitivity map. More extensive loss of macular sensitivity can be detected in this eye when compared to the younger patient. **f** Severely diminished/non-measurable electroretinogram recordings of photopic flash (top) and flicker stimulations (down), respectively. **g** Dark adaptometry illustrated the defective dark adaptation and extensively-prolonged rod intercept time in eye with CHM.

Central retinal sensitivity was assessed using the Macular Integrity Assessment (MAIA) microperimetry system (CenterVue SpA). The default “Expert Exam” mesopic and scotopic protocols were used. Mean sensitivity was recorded, and the colour-coded sensitivity map was exported (Figs. 2 and 3e).

Automated static perimetry was performed using the Octopus 900 perimeter (Haag-Streit Diagnostics). Sensitivity was measured at a custom 148-point grid covering 60° area. Tests with “Reliability Factor” greater than 33% were excluded. Mean sensitivity (MS), mean defect (MD), vertical and horizontal extent of the residual visual field (in degrees) parameters were collected for the purpose of statistical analysis (Fig. 2f).

### Dark adaptometry, colour testing, and handheld ERG

The AdaptDx dark adaptometer (MacuLogix) was used to measure dark adaptation. A bleaching photoflash (80% rod bleaching level) was used and testing duration was 13 min (Figs. 2 and 3, G). Rod intercept was calculated as the amount of time needed for the patient’s sensitivity to recover after bleaching to a stimulus intensity of 5 × 10^-3^ cd/m^2^ (3 log units). Rod intercept time longer than 6.5 min indicated dark adaptation dysfunction [17].

The Cambridge Colour Test v2.31 (Cambridge Research Systems Ltd) was used to assess the colour vision deficiency. The basic “Trivector” test version was performed to estimate discrimination thresholds along the protan, deutan, and tritan axes. Measurements exceeding 100 × 10^−3^ *u*′ *v*′ units for the protan and deutan vectors, and those exceeding 150 × 10^−3^ *u*′ *v*′ units in the tritan vector were considered abnormal.

Photopic flash (2 Hz) and flicker (30 Hz) ERGs were recorded using the handheld RETeval full-field ERG device (LKC Technologies, Inc.). White flash stimuli were presented to natural pupils and periorbital skin surface electrodes were used to record the ERG.

### Visual function questionnaire

Vision-related quality of life was assessed by using the 25-item National Eye Institute Visual Function Questionnaire (NEI VFQ-25) [18]. Patients are asked to rate the level of severity of visual symptoms or difficulty of daily tasks on a Likert scale. Scores in each subsection ranged from 0-100, with higher scores indicating better visual function and subsequent vision-related quality of life.

### Statistical analysis

Statistical analysis was performed using SPSS v. 25.0 (IBM Corporation), Microsoft Excel 2017 (Microsoft Corporation), and R. All measurements were reported as population mean ± standard deviation (SD) after filling in the missing longitudinal data using the last observation carry‐forward method [19]. Intra- and inter-grader variabilities were measured using coefficient of variation (CV), and intra-class correlation coefficient (ICC) with 95% confidence intervals (CI). CV was defined as the ratio between pooled standard deviation of the repeated measurements and the population mean. Bland-Altman plots were used to demonstrate the level of agreement between repeated measurements. Cross-sectional comparisons between the 2 groups of patients were assessed by Mann–Whitney U test or generalised estimating equations (GEE). GEE account for the intra-subject correlations between both eyes. Changes in areas of preserved EZ and CC over the 1 year follow up period were investigated using Wilcoxon signed-rank test. Related-samples Friedman’s two-way analysis of variance by ranks test was used to assess progressive changes in structural and functional parameters over the time of the 3 study-visits. If statistically significant difference was found using Friedman test, Dunn’s pairwise post hoc test was used to confirm where the differences occurred between visits. Holm–Bonferroni method was used to adjust *p* values for multiple comparisons. The correlation between age and structural and functional parameters were investigated using Spearman’s rank correlation coefficient (*rho*) and scatter plots. Pairwise partial correlations between structural and functional measurements were calculated while controlling for age (*r*) [20].

## Results

### Patients’ characteristics and clinical data

Thirteen male CHM patients (mean age ± SD, 45.08 ± 16.27 years; range, 20–74 years) from 8 pedigrees were included in this study. Twelve patients completed the 6-month follow up visit, and eleven participants completed the 1-year follow up visit. Genetic variants for study participants are reported in Supplemental Table S1. Characteristics of the enroled patients and their clinical data over the 1 year follow up time are summarised in Table 1. Eight patients were included in group 1 (mean age ± SD, 35 ± 10.1 years, median age, 34.5 years) and five patients were included in group 2 (mean age ± SD, 61.2 ± 8.2 years, median age, 62 years).

**Table 1:** Patients characteristics, clinical data, and self-reported vision-related quality of life.

	All Patients	Group 1	Group 2	Group 1 vs Group 2 (*p* value)
Participants	13	8	5	
Number of families	10	7	4	
Gender				
Female	0	0	0	
Male	13	8	5	
Age at baseline	45.08 ± 16.27	35.00 ± 10.09	61.20 ± 8.18	
Range	20–74	20–49	50–74	
Self-reported visual function	9	6	3	
General health	72.2 ± 23.2	79.2 ± 24.6	58.3 ± 14.4	0.26
General vision	51.1 ± 24.7	60.0 ± 25.3	33.3 ± 11.5	0.17
Ocular pain	95.8 ± 8.8	100.0 ± 0.0	87.5 ± 12.5	0.17
Near activities	64.8 ± 26.6	70.8 ± 29.7	52.8 ± 17.3	0.26
Distance activities	51.9 ± 21.6	54.2 ± 23.4	47.2 ± 21.0	0.55
Social functioning	61.1 ± 25.3	64.6 ± 26.7	54.2 ± 26.0	0.55
Mental health	59.0 ± 33.1	62.5 ± 27.1	52.1 ± 13.0	0.71
Role difficulties	58.3 ± 33.1	60.4 ± 34.8	54.2 ± 36.1	0.71
Dependency	77.8 ± 25.0	73.6 ± 30.5	86.1 ± 4.8	0.91
Driving	NA	NA	NA	NA
Colour vision	83.3 ± 33.1	87.5 ± 30.6	75.0 ± 43.3	0.71
Peripheral vision	41.7 ± 17.7	45.8 ± 18.8	33.3 ± 14.4	0.38
Average	64.8 ± 19.0	68.4 ± 20.4	57.6 ± 16.9	0.38
Eyes	26	16	10	
ETDRS letters	26	16	10	
Baseline	71.7 ± 23.9	82.00 ± 7.80	55.20 ± 31.61	0.006
6-month	70.6 ± 24.1	82.13 ± 7.84	52.20 ± 30.07	
1-year	70.4 ± 24.6	82.25 ± 8.31	51.30 ± 30.13	
LogMAR equivalent	25	16	9	
Baseline	0.28 ± 0.62	0.08 ± 0.16	0.62 ± 0.94	0.06
6-month	0.30 ± 0.61	0.08 ± 0.15	0.69 ± 0.90	
1-year	0.30 ± 0.62	0.07 ± 0.15	0.72 ± 0.90	
LogCS	26	16	10	
Baseline	1.48 ± 0.58	1.81 ± 0.20	1.20 ± 0.34	<0.001
6-month	1.46 ± 0.61	1.82 ± 0.16	1.14 ± 0.50	
1-year	1.45 ± 0.57	1.79 ± 0.18	1.01 ± 0.50	

Values are equal to N or population mean ± standard deviation. *P* values were calculated for the baseline visit and are based on Mann–Whitney U test or generalised estimating equations (GEE, accounting for the within-subject correlation between the two eyes, whenever needed).

*NA* not applicable, *ETDRS* early treatment diabetic retinopathy study, *LogMAR* logarithm of the minimum angle of resolution, *LogCS* logarithm of contrast sensitivity.

Central vision is generally preserved in patients with choroideremia, especially during early stages of the disease. At baseline, all eyes were able to see at least 55 letters on the ETDRS chart, except three eyes from three different patients (in group 2) which had a BCVA of 42 letters, hand motion, and light perception, with no apparent cause other than the choroideremia. In group 1, eyes were able to see on average 82.0 ± 7.8 letters on the ETDRS chart (equivalent to Snellen 6/7.5), whereas in group 2 it was 55.2 ± 31.6 letters (Snellen 6/24). The differences were found to be statistically significant between groups (*p* = 0.006, GEE). Similarly, CS was significantly worse in group 2 (*p* < 0.001, GEE).

**Fig. 4:**
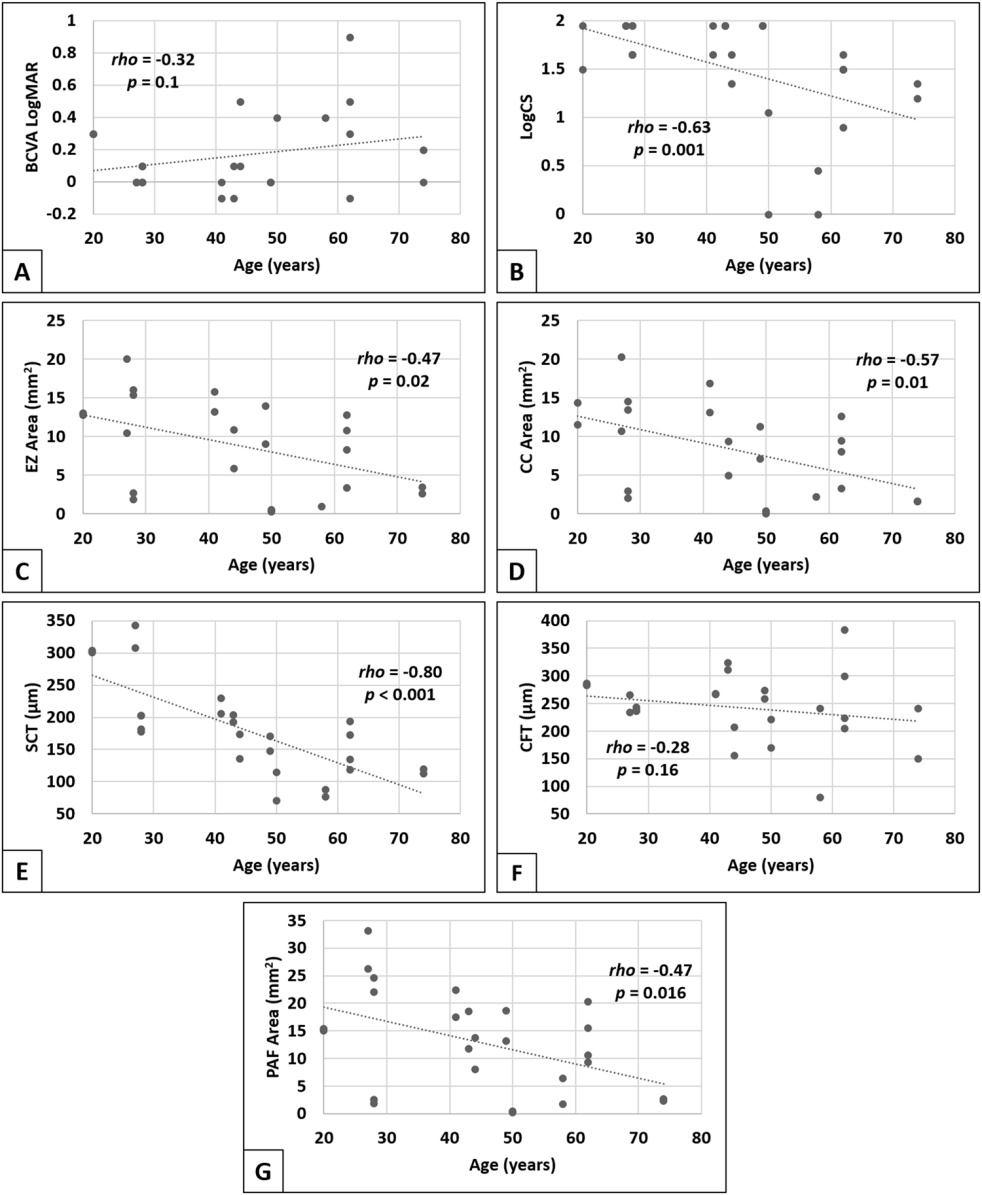
Correlations between structural and functional parameters, and age. Correlations were assessed by Spearman’s rank correlation coefficient (*rho*). BCVA best-corrected visual acuity, LogMAR logarithm of the minimum angle of resolution, LogCS logarithm of contrast sensitivity, EZ ellipsoid zone, CC choriocapillaris, SCT subfoveal choroidal thickness, CFT central foveal thickness, PAF preserved autofluorescence.

We observed a trend of decreasing VA and CS with increasing patient’s age (Fig. 4a, b); the correlation with CS was statistically significant (*rho* = −0.63, *p* = 0.001) but not so for VA (*rho* = −0.32, *p* = 0.1). Longitudinally, neither visual acuity nor contrast sensitivity showed significant progression over the 1-year follow up period within group 1 (*p* = 0.84 and 0.49, respectively, Friedman test) or group 2 (*p* = 0.20 and 0.34, respectively, Friedman test).

### Optical coherence tomography angiography

Twenty-three eyes from 12 patients had gradable OCTA scans at the baseline visit, with 16 eyes from 9 of these patients at the 1-year visit. *En face* OCTA of the choroid showed marked peripheral degeneration of CC, as well as the attenuation of the deeper large choroidal vessels (Figs. 2 and 3a). An island of relatively preserved CC was detected in all CHM eyes. Meanwhile, the *en face* OCT slab of EZ was able to provide a clear visualisation of EZ area. The preserved EZ island was characterised by a hyperreflective edge and surrounded by a dark hyporeflective “halo”. Outer retinal tubulations (ORT) were also clearly detected at the transitional zone between the degenerated and relatively preserved photoreceptor regions in all eyes (Fig. 1, yellow arrows in A1 and A2). Reliability analyses of EZ and CC measurements were reported in the Supplemental Information, Supplemental Table S2, and Supplemental Fig. S1.

At baseline visit, patients in group 1 had significantly larger areas of preserved CC and EZ compared to group 2 (*p* = 0.01 and 0.005, respectively, GEE) (Table 2). Ten eyes from group 1 and 6 eyes from group 2 had OCTA scans at the 1-year follow up time-point. Eyes in both groups showed a tendency of progressive constriction of the area of preserved CC and EZ. The 1-year rate of change in CC and EZ was found to be relatively consistent and statistically significant in younger patients (−10.0% and −7.9%, *p* = 0.009 and 0.01, respectively, Wilcoxon signed-rank test). Meanwhile, longitudinal changes in group 2 were not statistically significant (*p* = 0.12 and 0.06, respectively). However, the percentage rates of change in CC and EZ areas were statistically equivalent in both groups (*p* = 0.85 and 0.43, respectively, GEE). The preserved areas of CC and EZ at baseline showed moderate negative correlation with patient’s age (*rho* = −0.57 and −0.47, *p* = 0.01 and 0.02, respectively) (Fig. 4c, d).

**Table 2:** Structural and functional measurements in patients with choroideremia.

	All Patients	Group 1	Group 2	Group 1 vs Group 2 (*p* value at baseline)
Optical coherence tomography angiography (mm^2^)				
Preserved choroicapillaris area (*n*)	23	14	9	
Baseline	8.31 ± 5.85	10.87 ± 5.23	4.32 ± 4.53	0.01
1-year	7.96 ± 5.76	10.38 ± 5.21	4.19 ± 4.55	
Preserved ellipsoid zone area (*n*)	23	14	9	
Baseline	8.84 ± 5.92	11.47 ± 5.19	4.75 ± 4.66	0.005
1-year	8.39 ± 5.75	10.93 ± 5.12	4.44 ± 4.40	
Optical coherence tomography (μm)				
Central foveal thickness (*n*)	26	16	10	
Baseline	242.3 ± 60.7	255.4 ± 40.0	221.2 ± 82.3	0.18
6-month	241.4 ± 62.6	253.4 ± 46.7	222.1 ± 81.1	
1-year	236.1 ± 65.6	249.3 ± 49.3	215.1 ± 84.3	
Central choroidal thickness (*n*)	26	16	10	
Baseline	179.7 ± 72.6	217.3 ± 62.7	119.5 ± 39.2	<0.001
6-month	172.7 ± 73.3	209.7 ± 65.0	113.4 ± 39.2	
1-year	166.2 ± 73.3	202.6 ± 66.8	108.1 ± 37.4	
Fundus autofluorescence (mm^2^)				
Preserved autofluorescence area (*n*)	26	16	10	
Baseline	12.85 ± 9.03	16.55 ± 8.34	6.95 ± 6.89	0.01
6-month	12.13 ± 8.71	15.53 ± 8.24	6.69 ± 6.63	
1-year	11.64 ± 8.49	14.94 ± 8.19	6.35 ± 6.16	
Microperimetry (dB)				
Mesopic sensitivity (*n*)	25	16	9	
Baseline	10.5 ± 7.2	13.6 ± 6.5	5.0 ± 4.5	<0.001
6-month	10.5 ± 7.4	13.4 ± 6.8	5.4 ± 5.7	
1-year	10.9 ± 6.7	13.9 ± 5.6	5.6 ± 5.7	
Scotopic cyan sensitivity (*n*)	23	16	7	
Baseline	3.8 ± 4.6	5.2 ± 4.8	0.5 ± 0.9	0.02
6-month	1.8 ± 2.3	2.4 ± 2.5	0.4 ± 0.6	
1-year	1.7 ± 2.1	2.1 ± 2.4	0.7 ± 0.9	
Scotopic red sensitivity (*n*)	23	16	7	
Baseline	4.8 ± 4.9	6.3 ± 5.1	1.2 ± 1.7	0.006
6-month	3.8 ± 3.2	4.8 ± 3.3	1.6 ± 1.9	
1-year	3.4 ± 3.8	4.1 ± 4.2	1.7 ± 2.2	
Visual field				
Automated static perimetry (*n*)	17	11	6	
Mean sensitivity (dB)	3.6 ± 2.4	4.8 ± 2.1	1.3 ± 0.8	0.001
Mean defect (dB)	24.1 ± 2.0	23.4 ± 2.1	25.3 ± 0.7	0.58
Vertical extent (degrees)	22.0 ± 13.6	27.9 ± 12.8	11.2 ± 7.0	0.02
Horizontal extent (degrees)	10.9 ± 5.7	12.5 ± 6.1	7.8 ± 3.6	0.07
Trivector colour testing (× 10^-3^ u′ v′ units)				
Protan (*n*)	22	16	6	
Baseline	219.9 ± 192.8	154.1 ± 110.7	395.2 ± 261.9	<0.001
1-year	206.1 ± 192.9	142.4 ± 117.1	376.0 ± 259.8	
Deutan (*n*)	22	16	6	
Baseline	215.2 ± 184.4	146.8 ± 99.2	397.7 ± 438.0	<0.001
1-year	207.8 ± 176.1	154.3 ± 107.8	350.5 ± 248.4	
Tritan (*n*)	22	16	6	
Baseline	562.9 ± 377.8	421.5 ± 240.2	939.8 ± 438.0	<0.001
1-year	558.0 ± 327.3	446.1 ± 237.6	856.3 ± 365.5	

Values are equal to *n* (number of eyes) or population mean ± standard deviation. *P* values were calculated for the cross-sectional comparisons at baseline visit and are based on generalised estimating equations (GEE, accounting for the within-subject correlation between the two eyes).

### Optical coherence tomography

Central foveal (CFT) and subfoveal choroidal thickness (SCT) measurements at baseline, 6-month, and 1-year visits are summarised in Table 2. One patient (20 years old) showed evidence of bilateral epiretinal membrane with macular traction. Cystoid macular oedema (CMO) was detected in 15 eyes (57.5%) from 8 subjects (61.5%). The cysts were characteristically detected at the parafoveal area of the inner nuclear layer (INL), just beyond the edge of the central retained island of retinal tissue, specifically sparing the centre of the fovea (Fig. 3d). No differences were observed in the prevalence of CMO in groups 1 or 2, and there was no significant change in VA and CS due to CMO (*p* = 0.36 and 0.29, respectively, GEE).

At baseline, no statistically significant difference was observed between groups 1 and 2 for CFT (*p* = 0.18, GEE). Patients in group 2 showed marked thinning of mean SCT by 45% when compared to group 1 (*p* < 0.001, GEE). Over the 1-year period, slow but relatively consistent progressive thinning was detected in the central fovea in patients of both groups (Table 2). The changes over 6-month periods were not large enough to reach the significance level (*p* ≥ 0.08, Dunn’s pairwise post hoc tests). But between baseline and 1-year measurements, the 3.3% and 6.7% foveal thinning in groups 1 and 2, respectively, were found to be statistically significant (*p* = 0.006 and *p* = 0.003, Dunn’s post hoc test). The rate of retinal thinning appeared to be slightly faster in older patients, but the difference in percentage progression between both groups was not significant (*p* = 0.53, Wilcoxon signed-rank test).

The SCT showed progressive loss, with the rate of choroidal thinning being greater than central foveal tissue loss (*p* = 0.02, Wilcoxon signed-rank test). Both groups showed statistically significant differences in SCT measurements between the 3 visits (*p* < 0.001, Friedman test). The rate of choroidal loss between all visits (6-month and 1-year intervals) was statistically significant (*p* < 0.05, Dunn’s post hoc tests). SCT showed a strong negative correlation with patient’s age (*rho* = −0.80, *p* < 0.001) (Fig. 4e). However, no association was detected between CFT and age (*rho* = −0.28, *p* = 0.16) (Fig. 4f).

### Fundus autofluorescence

Patients with CHM typically show progressive concentric loss of autofluorescence, retaining an island of preserved AF (PAF) at the macula. Areas of PAF tended to be vertically expanded, and favouring the central and the temporal side of the macula (Figs. 2 and 3c). However, the progressively degenerating outer retina had already encroached upon the foveal centre at baseline in 6 eyes from 4 patients (44–62 years old), whereas the areas of PAF were detected at the adjacent parafoveal region.

Similar to other structural parameters, the average PAF in group 2 was significantly smaller than group 1 at baseline (*p* = 0.01, GEE), with statistically significant progressive shrinkage of PAF between scans 1-year apart (−9.9%, *p* < 0.001) and at 6-month intervals (*p* < 0.05). The absolute rate of PAF area reduction in mm^2^ was significantly larger in group 1 when compared to group 2 (−1.84 and −0.60 mm^2^/year, respectively, *p* = 0.008, GEE). However, the percentage rate of progression was statistically similar between both (−11.3% and −8.0%, *p* = 0.19, GEE). A moderate negative correlation was observed between patient’s age and the area of PAF (*rho* = −0.47, *p* = 0.016) (Fig. 4g).

### Microperimetry

Nineteen eyes from 12 patients had reliable mesopic and scotopic microperimetry testing during the baseline visit. All eyes had cone and rod system dysfunction, with the rod-mediated measurements being more severely affected.

Group 1 had significantly better mesopic function when compared to group 2 (*p* < 0.001, GEE). Similarly, in dark-adapted conditions, group 1 had significantly higher sensitivity to cyan and red stimuli (*p* = 0.02 and 0.006, respectively, GEE) (Table 2). Longitudinally, changes in mesopic and scotopic mean sensitivity were small and inconsistent during the 6-month and 1-year follow-ups (*p* > 0.05, Friedman tests).

The baseline mesopic sensitivity correlated moderately with age (*rho* = −0.53, *p* = 0.017). Meanwhile, scotopic sensitivity to cyan and red stimuli were only weekly and insignificantly associated with age at baseline (*rho* = −0.37 and −0.33, *p* > 0.1).

### Dark adaptation

Twenty eyes from 10 subjects underwent reliable dark adaptometry at least once during the study period. Unreliable tests with “Fixation Loss Rate” >30% were discarded. Severely affected eyes were unable to see the flashing stimulus, thus the test was not performed. Only one eye (age, 49 years) in our cohort had a normal rod intercept time of 5.34 min (normal: 6.5 min) (Fig. 2g). The DA testing was repeated after 1 year, showing significant prolongation in rod intercept (8.37 min) compared to baseline. The other eye of the same patient showed abnormal rod intercept times (>20 min) at baseline and 1-year testing sessions. Abnormal DA function was also detected in all other patients, with measured rod intercept time longer than 20 min.

### Colour testing

Eleven patients (22 eyes) underwent the colour testing at baseline and/or 1-year follow-up visit. All patients showed some degree of colour vision impairment, especially for the blue-yellow spectrum. Higher chromatic discrimination thresholds were observed in group 2 compared to group 1 in the protan, deutan, and tritan axes (*p* < 0.001, GEE) (Table 2). At baseline, abnormal colour discrimination in tritan axis was found in 17/18 (94%) of the eyes, and borderline sensitivity loss (141, normal: up to 150) was noted in the remaining eye. Meanwhile, less patients showed protan and deutan dysfunction (12 eyes, 67%). Sixteen eyes had colour testing at both the baseline and 1-year visits. No significant progression was observed over the 1-year period in any of the three vectors (*p* > 0.29, Wilcoxon signed-rank test). Similarly, we did not observe significant associations between age and baseline colour sensitivities in our cohort of patients with CHM (*rho* < 0.24, *p* > 0.34, in all three axes).

### Handheld electroretinography

The handheld ERG was performed in 16 eyes of 8 participants. The testing was robust and well-tolerated by patients, but the response to photopic flash and the 30-Hz flickers were either extensively weakened and delayed, or not measurable (Fig. 3f).

### Self-reported visual function

Nine patients completed the NEI VFQ-25 at the 1-year follow-up visit. All patients with CHM had impaired vision-related quality of life, with peripheral vision and general vision being the worst-affected modalities (Table 1). Group 2, with 3 patients who completed the NEI VFQ-25, had relatively worse vision-related quality of life compared to those in group 1. The worst-affected domain in the younger age-group was peripheral vision, whilst both general vision and peripheral vision were equally adversely affected in the older age group. Although the scores were generally better in group 1, no statistical significance was detected, possibly due to the small sample size.

### Correlations between structural and functional parameters

The photoreceptors, RPE and choriocapillaris layers were closely related targets for the degeneration seen in CHM. The measured preserved areas of CC, EZ, and AF correlated very strongly with one another (*r* ≥ 0.9, *p* < 0.001). Additionally, the SCT showed moderate association with CC, EZ, and PAF areas (*r* ≥ 0.6, *p* < 0.005), whereas, the CFT was poorly associated with preserved retinal areas (*r* < 0.3, *p* > 0.2).

**Fig. 5:**
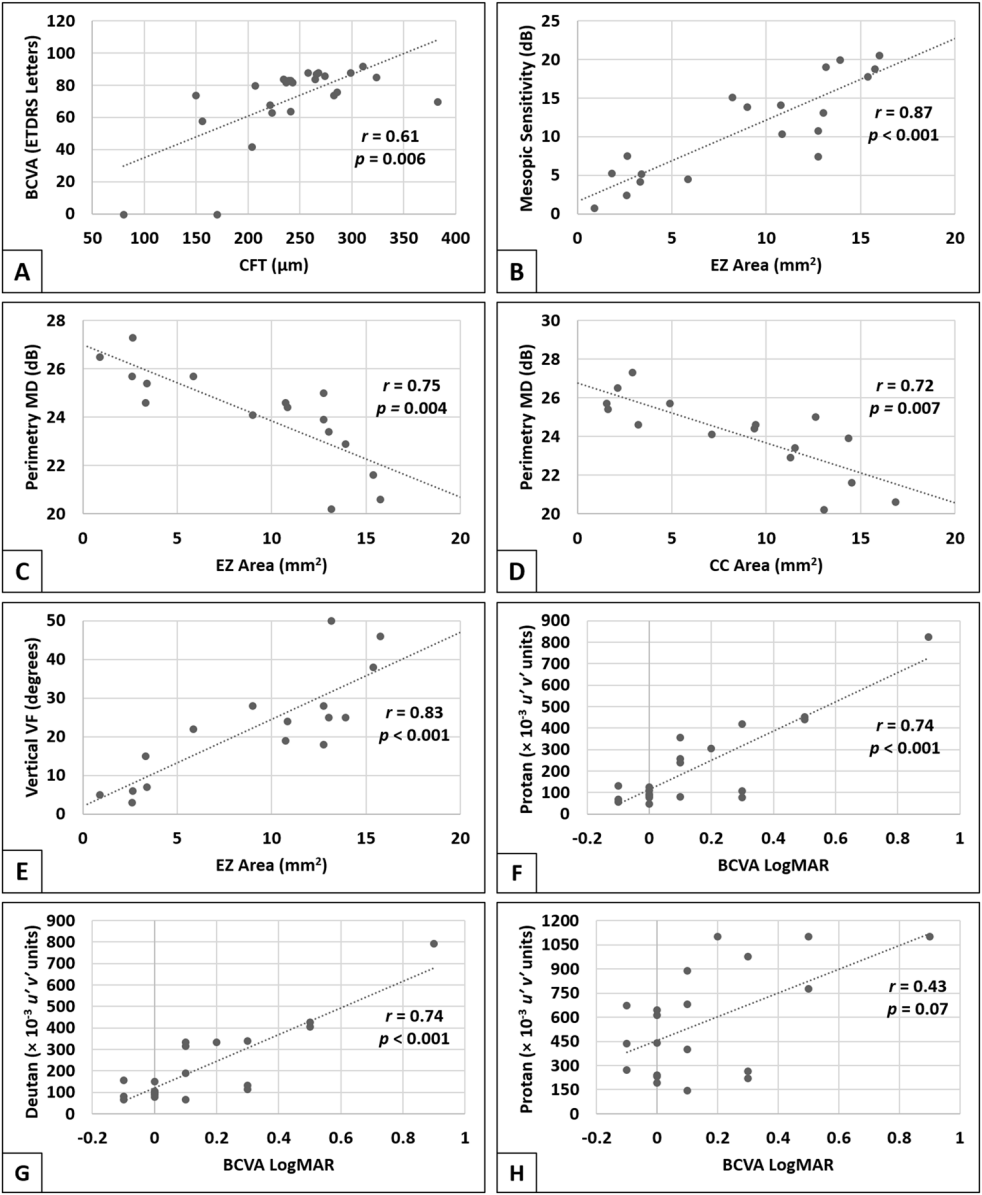
Pairwise partial correlations between structural and functional measurements. All correlations were controlled for age. *r*: partial correlation coefficient, BCVA best-corrected visual acuity, ETDRS early treatment diabetic retinopathy study, CFT central foveal thickness, EZ ellipsoid zone, MD mean defect, CC choriocapillaris, Vertical VF vertical extent of visual field, LogMAR logarithm of the minimum angle of resolution.

CFT was the only structural parameter which correlated with visual acuity (*r* = 0.61, *p* = 0.006) (Fig. 5a). The decline in contrast sensitivity had modest relationships with the structural changes in CHM including CFT and preserved areas of CC and outer retinal structures (*r* ≃ 0.5, *p* ≤ 0.025).

In microperimetry, the area of EZ was the largest determinant of mesopic macular sensitivity (*r* = 0.87, *p* < 0.001) (Fig. 5b). Area of CC and, to less extent, PAF were also significantly correlated with mesopic MS (*r* = 0.81 and 0.60, *p* = 0.001 and 0.02, respectively). The CFT and SCT were not associated with mesopic or scotopic sensitivity (to cyan and red stimuli) in our cohort of patients.

Visual field parameters were investigated for the relationship with retinal structure. MD was significantly negatively correlated with preserved areas of CC and EZ (*r* = 0.72 and 0.75, *p* = 0.007 and 0.004, respectively) (Fig. 5c, d), but not with PAF (*r* = −0.41, *p* = 0.08). The vertical extent of the preserved VF was most strongly related to the area of the EZ (*r* = 0.83, *p* < 0.001) (Fig. 5e), and to a lesser extent with the RPE and CC areas (*r* = 0.69 and 0.76, respectively, *p* = 0.003).

For colour vision, a significant correlation was detected between the protan and deutan colour discrimination and the area of the preserved EZ (*r* = −0.64 and −0.55, *p* = 0.016 and *p* = 0.04, respectively) and VA (*r* = 0.74, *p* < 0.001) (Fig. 5f, g). However, no associations were detected with the areas of CC or PAF. Tritan sensitivity did not correlate with any of the three structural parameters, nor VA (*r* = 0.43, *p* = 0.07) (Fig. 5h).

## Discussion

This longitudinal study provides detailed characterisation of structural and functional abnormalities in CHM patients. It is the first to describe the rate of structural loss in CHM within different age groups. CHM causes progressive concentric retinal and choroidal degeneration, with a characteristic preserved small island of retinal tissue at the macula until late stage of the disease. OCTA acquires simultaneous structural and angiographic data, allowing for the concurrent investigation of the outer retina and choroid [21–23]. In this paper, high contrast *en face* images were generated for direct visualisation with reliable delineation and measurement of areas of preserved EZ and CC. The definition of EZ and CC slabs were based only on BM segmentation, which is easier and less laborious to segment compared to the EZ line [24]. Although both measurements had excellent intra-grader and inter-grader ICC, the variability in EZ was 2.5 times lower than CC. This can be attributed to the fact that OCT angiograms are inherently more prone to noise and image artefact than structural OCT [25]. However, the mean annual loss rate was still larger than the coefficient of variation for EZ and CC area measurements, indicating the reliability of these methods in assessing the progression of structural defects in CHM. Significant progressive loss of the central retinal island was observed in patients <50 years old, but not in those ≥50 years of age. The rate of loss of PAF was also slower in ≥50 years patients, suggesting reduced disease progression with age. Previous cross-sectional studies reported logarithmic relationship between PAF and age [12, 26], which indicates slower structural changes in older patients.

Although, EZ, PAF islands and CC had very strong correlations between their area measurements, the temporal relationship between photoreceptors, RPE and CC loss is controversial. In our study, CC and EZ images were produced from the same scan, thus we were able to perform direct comparisons between these areas. But as FAF imaging covers a larger area than OCTA, direct comparison between PAF and CC/EZ area measurements was not possible. Nearly 70% of eyes showed more degeneration in the CC than the EZ. Additionally, a steady decrease in choroidal thickness was observed at the foveal centre of all patients, which long preceded any observable central changes on OCT or FAF. These findings suggest that photoreceptor and RPE loss are secondary to the vascular changes in the CC and/or deeper choroidal vessels. Histopathologic studies previously suggested a primary vascular problem in CHM due to defects in vascular endothelial cells of the choroidal vessels [27, 28]. Additionally, CHM is known to start at the equator which corresponds to the choroidal watershed zone [2], supporting a significant vascular component in the primary pathophysiology of CHM.

Cystic macular oedema was a common feature in our cohort. The prevalence of unilateral and bilateral CMO in our cohort (61.5% and 53.8%, respectively) are very similar to previous reports of 62.5% and 50.0%, respectively [29]. However, unlike their findings, no visual loss was associated with the presence of CMO in our cohort of CHM patients. Two patients with CMO have previously been treated with topical dorzolamide, all showed a marked improvement in macular thickness, but only 1 eye showed some improvement in VA [30]. This raises suspicions on the actual effect of CMO on visual function in CHM. Further studies are needed to investigate the impact of CMO and treatment efficacy on VA in CHM.

The preserved retinal island generally allows patients to retain good VA and CS until the degeneration reaches their fixation centre, usually after the fourth or fifth decade of life. In our cohort, patients with poorest VA and CS were older (≥ 50 years) and exhibited severe degenerative changes at the fovea, forcing them to use the relatively preserved adjacent parafoveal regions for fixation. These findings are consistent with VA findings from previous reports [13, 31]. We were not able to detect a direct relationship between VA and age as assessed by Spearman’s correlation. However, a retrospective cross-sectional study by Seitz and colleagues showed exponential association between age and VA [26]. Reductions in CS were previously observed in a series of 2 patients with CHM and CMO [30]. However, to the best of our knowledge, our paper is the first to investigate the correlation between age and LogCS. Abnormalities in CS have been linked not only to dysfunction of the retina [32], but also at different levels of the visual pathway up to the visual cortex [33].

Our investigations of the colour discrimination in our cohort revealed higher prevalence of tritan deficiency (blue colour) compared to protan-deutan (red-green colours). The blue colour discrimination is mediated by S-cones which are most prevalent in the parafoveal region, while the red and green colours discrimination is thought to be dependent on the L- and M-cones, with highest density at the centre of the fovea. The spatial distribution of different classes of cone photoreceptors might provide an explanation for the variation in patients’ perception sensitivity to different colours. The relative preservation of the central retinal island can retain more L- and M-cones than S-cones, maintaining the red-green discrimination until more advanced disease stages. The observed correlation between red-green sensitivity and VA also agree with the aforementioned explanation since VA is also dependent on the cones at the centre of the fovea. Meanwhile, this correlation was not observed with tritan discrimination. A recent study by Seitz et al. reported similar results by using the “ellipses” test from the Cambridge Colour Test [34]. By contrast, Jolly and colleagues used the Farnsworth Munsell 100 Hue test and observed generalised colour discrimination defects in their cohort of CHM patients, with no preferential loss in blue or red-green sensitivity [35]. Several earlier reports also investigated colour vision in CHM with mostly unspecified, qualitative, and variable results [14, 36, 37]. The contradicting results in literature can be attributed to the wide variety of colour tests used and the differences in patient characteristics between studies.

Visual field and mesopic microperimetry showed marked constriction, with the area of preserved sensitivity closely following the extension of the retained retinal tissue, with no apparent residual function outside the central retinal island. Jolly and colleagues reported exponential relationship between mean sensitivity and age [16], which supports our findings of significant correlation on Spearman’s *rho*. However, we did not observe significant differences on the longitudinal data from microperimetry testing. Previous microperimetry reliability studies in CHM patients revealed high inter-test variability, especially at the borders of the atrophic tissue [38]. Fatigue, learning curve, and intrinsic pathological processes are some of the factors that might produce some degree of test-retest variability.

With the advent of several therapeutic approaches for CHM [7, 39, 40], detailed phenotypic and genotypic characterisation has become a necessity. Structural parameters including subfoveal choroidal thickness as well as areas of preserved EZ, CC, and PAF provide reliable quantitative measurements for clinical trials. They show strong correlation with age and they are sensitive enough to detect minor longitudinal changes over the 1-year follow-up period. In contrast, functional parameters show significant correlations with patient age but no reliable change over 1-year. Slow functional progression, visit-to-visit variability, or the subjective nature of these tests may limit applicability of these parameters to detect short-term changes in CHM.

The main limitations of this study were a relatively small sample size with only adult participants and a relatively short period of follow-up time (1 year). Given the slow-progressive nature of CHM, longer follow-up with representation from the paediatric population would confirm the findings of this study and provide more insight into the earlier changes seen, guiding optimal intervention age. We only recruited participants retaining some degree of visual function to be able to comply with study procedures. This can potentially produce a selection bias by excluding severely affected patients. However, in our cohort, we had several eyes with advanced CHM with central degeneration and severe visual impairment. Another limitation of the study is the utility of manual segmentation of BM and areas of CC, EZ, and PAF which can be relatively time consuming and prone to intra- and inter-grader variability. The use of advanced image processing and machine learning techniques might enhance the visualisation and automated analysis of ophthalmic images [41–44].

In conclusion, longitudinal multimodal assessment of patients with CHM provides valuable information on the natural history of structural and functional status of the degenerating eyes. Structural parameters appear more sensitive for subtle short-term changes. Detailed correlation analyses between retinochoroidal structure and function can give some insight into the pathophysiological processes. Further studies are needed for better characterisation of the progression patterns in patients with CHM.

### Summary

#### What was known before

- Previous cross-sectional and retrospective studies suggested progressive degeneration of the RPE and photoreceptors.
- Emerging clinical trials for the treatment of choroideremia require the identification of proper metrics to monitor disease progression and response to treatment.

#### What this study adds

- The findings of this prospective longitudinal study suggest that vascular choroidal abnormalities precede apparent photoreceptor loss in choroideremia.
- Structural parameters appear more sensitive for monitoring subtle short-term changes in choroideremia.

## Supplementary information

Supplementary Information

Supplementary Table S1

Supplementary Table S2

Supplementary Figure S1
