# Supplementary material for: Prospective deep phenotyping of choroideremia patients using multimodal structure-function approaches
Source: Eye (Lond). 2020 May 28;35(3):838–52. doi: 10.1038/s41433-020-0974-1 (PMC8027673; doi:10.1038/s41433-020-0974-1)
Supplement: Supplementary file 1 — Supplementary Information [file 41433_2020_974_MOESM1_ESM.pdf]

Reliability of ellipsoid zone (EZ) and choriocapillaris (CC) area measurements

Measuring the areas of preserved EZ and CC from *en face* optical coherence tomography (OCT)/OCT angiography (OCTA) images showed good intra-grader and inter-grader reliability (Supplementary Table S2). Generally, the OCTA CC area had larger measurement variability than OCT EZ area as measured by CV and ICC. The agreement between gradings was also illustrated using Bland-Altman plots (Supplementary Figure S1). The charts demonstrate the relatively wider 95% limits of agreement in CC area measurements (Supplementary Figure S1, A and B) compared to the EZ area measurement (Supplementary Figure S1, C and D). Additionally, absolute difference between repeated measurements tended to be bigger in eyes with larger preserved EZ or CC areas (Supplementary Figure S1).
