# Supplementary material for: Prospective deep phenotyping of choroideremia patients using multimodal structure-function approaches
Source: Eye (Lond). 2020 May 28;35(3):838–52. doi: 10.1038/s41433-020-0974-1 (PMC8027673; doi:10.1038/s41433-020-0974-1)
Supplement: Supplementary file 2 — Supplementary Table S1 [file 41433_2020_974_MOESM2_ESM.pdf]

Supplementary Table S1. Genetic variants for choroideremia patients.			
**Subject**	**Pedigree**	**Mutation cDNA**	**Mutation Protein**
**Patient 001**	A	c.126C>G	p.Tyr42*
**Patient 003**	B	c.715C>T	p.Arg239*
**Patient 005**	C	c.1347C>G	p.Tyr449*
**Patient 006**	C	c.877C>T	p.Arg293*
**Patient 007**	D	c.698C>G	p.Ser233*
**Patient 010**	B	c.715C>T	p.Arg239*
**Patient 011**	E	c.715C>T	p.Arg239*
**Patient 013**	B	c.715C>T	p.Arg239*
**Patient 014**	F	c.757C>T	p.Arg253*
**Patient 015**	B	c.715C>T	p.Arg239*
**Patient 017**	G	c.715C>T	p.Arg239*
**Patient 018**	H	p.799C>T	p.Arg267*
**Patient 044**	E	c.715C>T	p.Arg2A39*
