# Supplementary material for: Prospective deep phenotyping of choroideremia patients using multimodal structure-function approaches
Source: Eye (Lond). 2020 May 28;35(3):838–52. doi: 10.1038/s41433-020-0974-1 (PMC8027673; doi:10.1038/s41433-020-0974-1)
Supplement: Supplementary file 3 — Supplementary Table S2 [file 41433_2020_974_MOESM3_ESM.pdf]

Supplementary Table S2. Reliability of ellipsoid zone and choriocapillaris area measurements			
		**EZ Area**	**CC Area**
**Grader 1**			
	**Measurement 1**	7.84 ± 5.49	7.34 ± 5.43
	**Measurement 2**	7.71 ± 5.45	7.22 ± 5.31
**Grader 2**		7.90 ± 5.45	7.45 ± 5.43
**Intra-Grader**			
	**Mean ± SD**	7.78 ± 5.47	7.28 ± 5.37
	**Difference**	0.13 ± 0.25	0.12 ± 0.63
	**Percentage Difference**	3.3%	8.6%
	**95% Limits of Agreement**	-0.37 – 0.64	-1.13 – 1.37
	**Pooled SD**	0.20	0.44
	**Coefficient of Variation**	2.5%	6.0%
	**ICC (95% CI)**	0.999 (0.998 – 1.000)	0.997 (0.992 – 0.999)
**Inter-Grader**			
	**Mean ± SD**	7.87 ± 5.47	7.39 ± 5.42
	**Difference**	0.06 ± 0.30	0.11 ± 0.74
	**Percentage Difference**	3.8%	10.0%
	**95% Limits of Agreement**	-0.66 – 0.54	-1.59 – 1.37
	**Pooled SD**	0.21	0.52
	**Coefficient of Variation**	2.7%	7.0%
	**ICC (95% CI)**	0.999 (0.998 – 1.000)	0.995 (0.989 – 0.998)

EZ: ellipsoid zone, CC: choriocapillaris, SD: standard deviation, ICC: intra-class correlation coefficient, CI: confidence interval
