# Supplementary figures and images for: Prospective deep phenotyping of choroideremia patients using multimodal structure-function approaches
Source: Eye (Lond). 2020 May 28;35(3):838–52. doi: 10.1038/s41433-020-0974-1 (PMC8027673; doi:10.1038/s41433-020-0974-1)

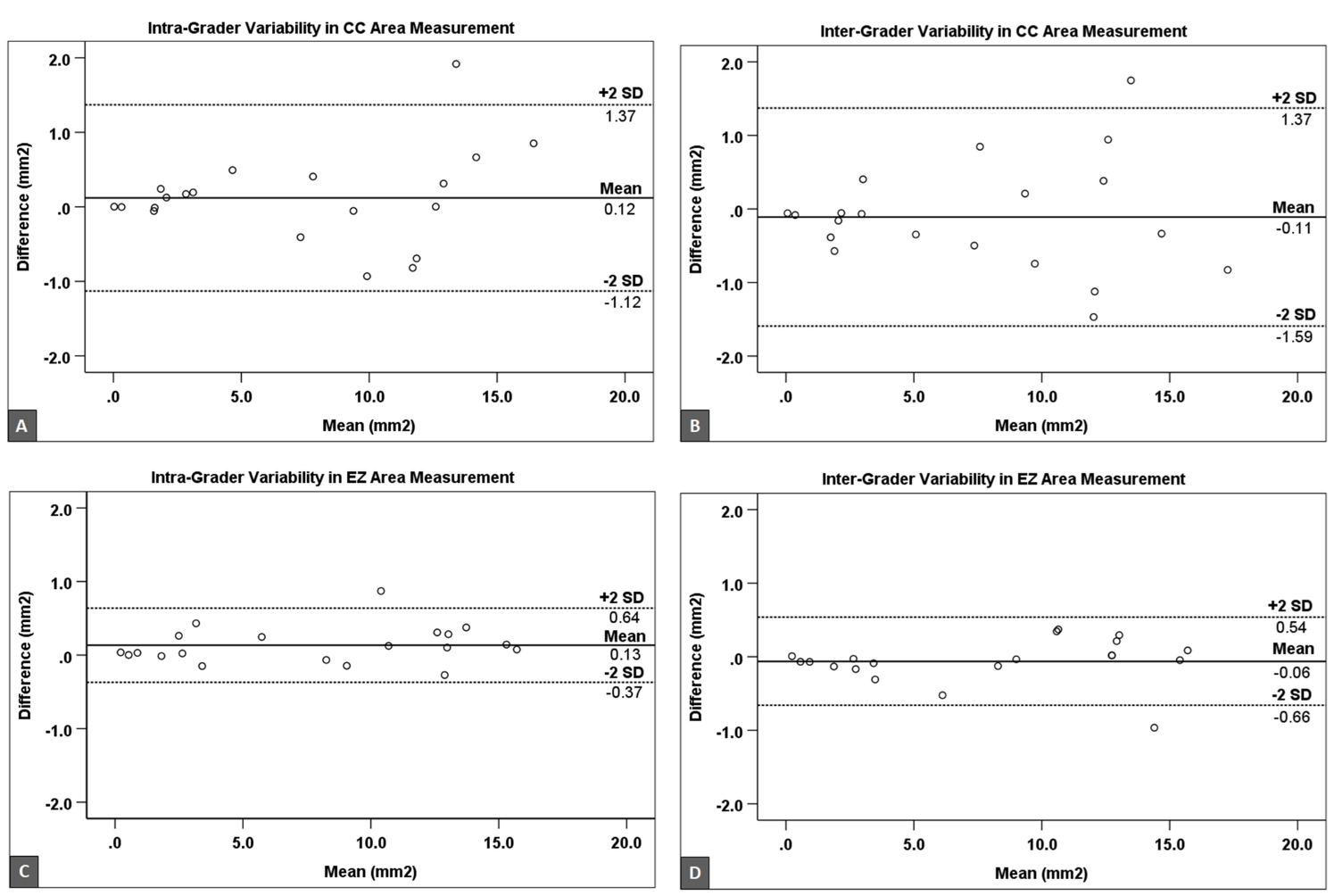

Supplement: Supplementary file 4 — Supplementary Figure S1 [file 41433_2020_974_MOESM4_ESM.tif]
